# Prevalence of HLA-B27, clinical characteristics and treatment outcomes in children with enthesitis-related arthritis

**DOI:** 10.1186/s12887-024-05032-2

**Published:** 2024-08-30

**Authors:** Boonsiri Jittawattanarat, Sirirat Charuvanij, Sirikarn Tangcheewinsirikul, Maynart Sukharomana

**Affiliations:** 1 Department of Pediatrics, Faculty of Medicine Siriraj Hospital, Mahidol University, Bangkok, Thailand; 2 Division of Rheumatology, Department of Pediatrics, Faculty of Medicine Siriraj Hospital, Mahidol University, 2 Wanglang Road, Bangkoknoi, Bangkok, 10700 Thailand; 3 Division of Rheumatology, Department of Pediatrics, Faculty of Medicine Vajira Hospital, Navamindradhiraj University, Bangkok, Thailand

**Keywords:** Enthesitis-related arthritis, HLA-B27, Clinical characteristics, JSpADA score, Treatment outcomes

## Abstract

**Background:**

Enthesitis-related arthritis (ERA) is a subtype of juvenile idiopathic arthritis with high disease burden. The objectives of this study were to explore the prevalence of HLA-B27, clinical characteristics, and treatment outcomes in children with ERA and compare the differences between HLA-B27 positive and negative patients.

**Methods:**

A retrospective cohort study at a pediatric rheumatology clinic in a tertiary referral hospital in Bangkok, Thailand, including ERA patients with at least 6 months of follow-up (July 2011-April 2022) was performed. Data were collected from medical records from diagnosis to recent follow-up, assessing disease activity and treatment outcomes, with an analysis comparing HLA-B27 positive and negative patients. Descriptive statistics were used for data analysis.

**Results:**

There were 59 ERA patients with mean age ± SD at diagnosis 11.2 ± 2.5 years, 53 males (89.8%), and positive HLA-B27 in 38 patients (64.4%). The HLA-B27 positive group had significantly higher levels of inflammatory markers at initial diagnosis (*p* = 0.001), lower baseline hemoglobin (*p* = 0.001) and hematocrit (*p* = 0.002), higher disease activity assessed by the Juvenile Spondyloarthritis Disease Activity score at 6 and 12 months of follow-up (*p* = 0.028 and 0.040, respectively), increased utilization of bridging systemic corticosteroids (60.5% vs. 14.3%, *p* = 0.001) and anti-TNF (39.5% vs. 9.5%, *p* = 0.018), and longer duration of methotrexate (median[IQR] 1.7[1.1–3.1] vs. 1.3[0.6–1.9] years, *p* = 0.040). The HLA-B27 negative group had more prevalent hip arthritis than the positive group at initial diagnosis (66.7% vs. 28.9%, *p* = 0.005) and during the course of the disease (71.4% vs. 36.8%, *p* = 0.011).

**Conclusion:**

Most of the ERA patients tested positive for HLA-B27. Throughout the follow-up period, these patients demonstrated greater disease activity, greater use of corticosteroids and anti-TNF, and longer duration of methotrexate to control the disease.

## Key points

• HLA-B27 was tested positive in nearly two-thirds of enthesitis-related arthritis (ERA) patients.

• ERA patients with positive HLA-B27 had significantly higher levels of inflammatory markers with lower hemoglobin and hematocrit at initial diagnosis, increased disease activity assessed by the Juvenile Spondyloarthritis Disease Activity (JSpADA) score at 6 and 12 months of follow-up, greater utilization of bridging systemic corticosteroids and anti-TNF, and longer duration of methotrexate use.

• ERA patients with negative HLA-B27 had more prevalent hip arthritis at initial diagnosis and during disease course.

• Application of the Assessment of SpondyloArthritis international Society (ASAS) classification for adult spondyloarthritis in ERA patients who entered adulthood showed the predominance of peripheral spondyloarthritis in more than half of the patients.

## Introduction

Juvenile idiopathic arthritis (JIA) is the most common cause of chronic arthritis in children, with an incident rate of 1.6–42.5/100,000 and a prevalence of 3.8–400/100,000 population [1, 2]. Enthesitis-related arthritis (ERA), one of the 7 subtypes classified according to the International League of Associations for Rheumatology (ILAR), can be classified by having arthritis and enthesitis or arthritis/enthesitis along with at least 2 of the following: sacroiliitis or inflammatory back pain, positive blood test for human leukocyte antigen (HLA)-B27, arthritis in males with onset age greater than 6 years, symptomatic anterior uveitis, and/or first-degree relative family history of HLA-B27-associated disease [1]. The incidence of ERA in Europe and the Middle East ranges from 5 to 15% of all cases of JIA in children [3, 4]. In Thailand, previous studies reported that ERA accounts for 21.5–33.7% of JIA patients [5–7]. Due to the chronicity of the disease associated with severe arthritis and enthesitis, the treatment of ERA has been more challenging than other subtypes of JIA subtypes [8, 9]. Less than 20% of patients with ERA would have inactive disease after 5 years of continuous treatment, and some may develop ankylosing spondylitis in 10 years, affecting morbidity and negatively affecting quality of life [7, 10–12]. Accordingly, early diagnosis and prompt intervention can enhance long-term treatment outcomes [7].

The treatment recommendation for ERA varies based on disease severity, including non-steroidal anti-inflammatory drugs (NSAIDs), disease-modifying antirheumatic drugs (DMARDs, e.g. methotrexate and sulfasalazine), corticosteroids (intra-articular injection as adjunctive therapy and oral prednisolone as bridging therapy in moderate and high disease activity), and biologics e.g. antitumor necrosis factor (anti-TNF) for severe and refractory patients having multiple active arthritis, enthesitis and sacroiliitis that do not respond to other treatment modalities [13]. In resource-limited countries, biologics prescription is limited due to high costs [13], and Thailand is one of the countries that may not provide it to all patients with refractory JIA because financial coverage depends on individual health benefit schemes and the fulfillment of the eligibility criteria for biologics in JIA, including refractory disease despite treatment with combinations of NSAIDs, DMARDs and systemic corticosteroids [7].

From the literature review, HLA-B27 positivity is recognized as a poor prognostic factor. The incidence of positive HLA-B27 varies from 43 to 91.8% in ERA patients [14]. Patients with positive HLA-B27 tend to be less responsive to standard therapy and need more anti-TNF to control the disease until remission [15–17]. Other poor prognostic factors have been proposed in previous studies, such as an age of onset of more than 8 years, hip arthritis, ankle arthritis within the first 6 months after diagnosis, inflammatory back pain, and a family history of ankylosing spondylitis [12, 17].

ERA encompasses diversity in clinical presentations, disease outcomes across different ethnicities, and country-specific access restrictions to biologics. Therefore, we seek to look more closely at the aforementioned aspects in relation to other global regions. The objectives of this study were to explore the prevalence of HLA-B27, clinical characteristics, disease activity, and treatment results in children with enthesitis-related arthritis (ERA), and to compare the differences between patients positive and negative for HLA-B27. This study aims to provide useful information to refine treatment practices within the country and globally.

## Materials and methods

This retrospective cohort study was conducted at the Department of Pediatrics, Faculty of Medicine Siriraj Hospital, Mahidol University, a tertiary referral medical center in Bangkok, Thailand. The data was acquired from July 2011 to April 2022 through a retrospective review of an electronic medical database recorded by pediatric rheumatologists. The study protocol was approved by the Ethics Committee for Research in Humans of the Siriraj Institutional Review Board of the Faculty of Medicine Siriraj Hospital, Mahidol University (Certificate of Approval number Si 359/2022). Due to the retrospective study design, the informed consent and assent were waived by the Siriraj Institutional Review Board.

Participants in this study were patients diagnosed with ERA according to ILAR classification criteria [1] with a minimum of 6 months of follow-up in the pediatric rheumatology clinic at Siriraj Hospital. In this study, we reviewed and collected data from medical records of follow-up visits by pediatric rheumatologists to assess clinical manifestations and disease activity during the treatment course in ERA patients, with a comparison between the HLA-B27 positive and negative groups. Baseline patient characteristics included sex, age at onset and diagnosis, family history of HLA-B27-related diseases in first-degree relatives, recent infection history, and clinical manifestations (e.g. number of active arthritis, active enthesitis, hip joint involvement, sacroiliitis, inflammatory back pain, limitation of joint range of motion, and acute symptomatic uveitis) were collected. Initial laboratory findings including complete blood count (CBC), erythrocyte sedimentation rate (ESR), C-reactive protein (CRP) and imaging studies were obtained. The HLA-B27 positivity was tested by using the microlymphocytotoxicity method [18]. We also documented individual patient medication usage with duration, adhering to standard recommendations for treatment of ERA [19, 20], consisting of NSAIDs, systemic corticosteroids, intra-articular corticosteroids injections, methotrexate (subcutaneous or oral), sulfasalazine, and anti-TNF agents (etanercept, adalimumab, or infliximab). In Thailand, the eligibility criteria for anti-TNF for JIA are considered based on individual health benefit schemes and are indicated in patients with refractory disease despite treatment with combinations of NSAIDs, DMARDs, and systemic corticosteroids. Therefore, at our center, all ERA patients received treatment combinations consisting of NSAIDs, conventional DMARDs, and systemic corticosteroids as a bridging therapy before addition of biological DMARDs.

To assess disease severity and monitor disease activity, the Juvenile Spondyloarthritis Disease Activity (JSpADA) score [19] was assessed at diagnosis, 6 months, 12 months, and at the most recent follow-up visit. The JSpADA score consists of 8 items: active joint count, active enthesitis count, visual analog pain scale (VAS, 0–10), ESR or CRP, morning stiffness (greater than 15 min), clinical sacroiliitis, uveitis, and limited back mobility; these data were recorded in the medical records during each visit by pediatric rheumatologists. Scores range from 0 to 8, assuming equal importance for each item, with higher scores indicating higher disease activity [19].

The status of the disease at the final follow-up was classified as active disease, inactive by clinical evaluation, inactive by Wallace criteria, remission on medication and remission without medication [20]. Furthermore, refractory or relapsed disease was observed during the follow-up period [16]. The definitions of disease status in this study were as follows:

- Inactive disease clinically was defined as the absence of active arthritis or uveitis, with the physician’s global assessment of disease activity indicating that there was no disease activity [20].
- Inactive disease according to Wallace criteria was met when the criteria for inactive disease was met by clinical measures and inflammatory markers (ESR or CRP) were within the normal range [20].
- Clinical remission was achieved when the inactive disease criteria was met by the Wallace criteria. This was further categorized into clinical remission with medication (continuous inactivity while on medication for at least 6 months) and clinical remission without medication (continuous inactivity for more than 12 months after discontinuing medication) [21].
- Refractory disease was defined as active arthritis that persisted despite treatment with at least two types of DMARDs for a minimum of 6 months or the presence of active enthesitis or sacroiliitis despite the use of at least one type of DMARD for at least 3 months [16].
- Relapse in disease was defined as the reappearance of active symptoms that did not meet the criteria for inactive disease after achieving clinical remission for at least 6 months on or off medications.
- Enthesitis was clinically diagnosed and defined as inflammation of the enthesis (insertion of tendons, ligaments and fascia), which can involve any site in the body.
- Sacroiliitis was clinically diagnosed and confirmed by magnetic resonance imaging at diagnosis and during the follow-up period.

In adults, the Assessment of SpondyloArthritis international Society (ASAS) classification criteria [22, 23] are used to assess spondyloarthritis (SpA), distinguishing between the predominant peripheral and axial forms. This study applied the ASAS classification to patients older than 16 years at their last visit.

All patients were clinically monitored during the follow-up period for the potential development of clinical manifestations for inflammatory bowel disease which has been reported to be associated with juvenile spondyloarthropathy and HLA-B27 positivity [19]. In cases with clinical index of suspicion for inflammatory bowel disease, pediatric gastroenterology specialists were consulted for further evaluation including esophagogastroduodenoscopy with colonoscopy and serology tests.

### Statistical analysis

The data collected were analyzed using IBM SPSS Statistics Version 28 (IBM Corp, Armonk, NY, USA). The determination of the sample size was calculated by the previous study conducted by Arkachaisri et al [14], by the formula n = Z^2^_ɑ/2_p(1-p)/*e*^2^ (defined type I error (α) = 0.05, allowable error (*e*) = 0.1, Z_ɑ/2_=Z_0.025_=1.96, *p* = 0.822) which resulted in a calculated sample size of a minimum of 57.

Descriptive statistics were used for the analysis. Categorical data were conveyed as frequencies with percentages. Continuous data were presented as either mean ± standard deviations (SD) for normally distributed data or median with interquartile range (IQR) for nonnormally distributed data. Comparison of differences between HLA-B27 positive and negative patients was analyzed using an independent samples t-test (for normally distributed data) and the Mann-Whitney U test (for nonnormally distributed data) for continuous variables, while categorical variables were evaluated via the chi-square test and Fisher’s exact test. Statistical significance was inferred for *p*-values less than 0.05. We adhered to the reporting guidelines described in Strengthening the Reporting of Observational Studies in Epidemiology [24].

## Results

In a cohort of 62 patients diagnosed with ERA from a total of 178 JIA patients who received care at the pediatric rheumatology outpatient clinic at Siriraj Hospital, 3 patients were excluded from the analysis due to a lack of sufficient follow-up duration of less than 6 months. Therefore, 59 patients were included in the analysis, all of whom had a follow-up duration of at least 6 months.

### Baseline clinical characteristics at diagnosis

**Fig. 1:**
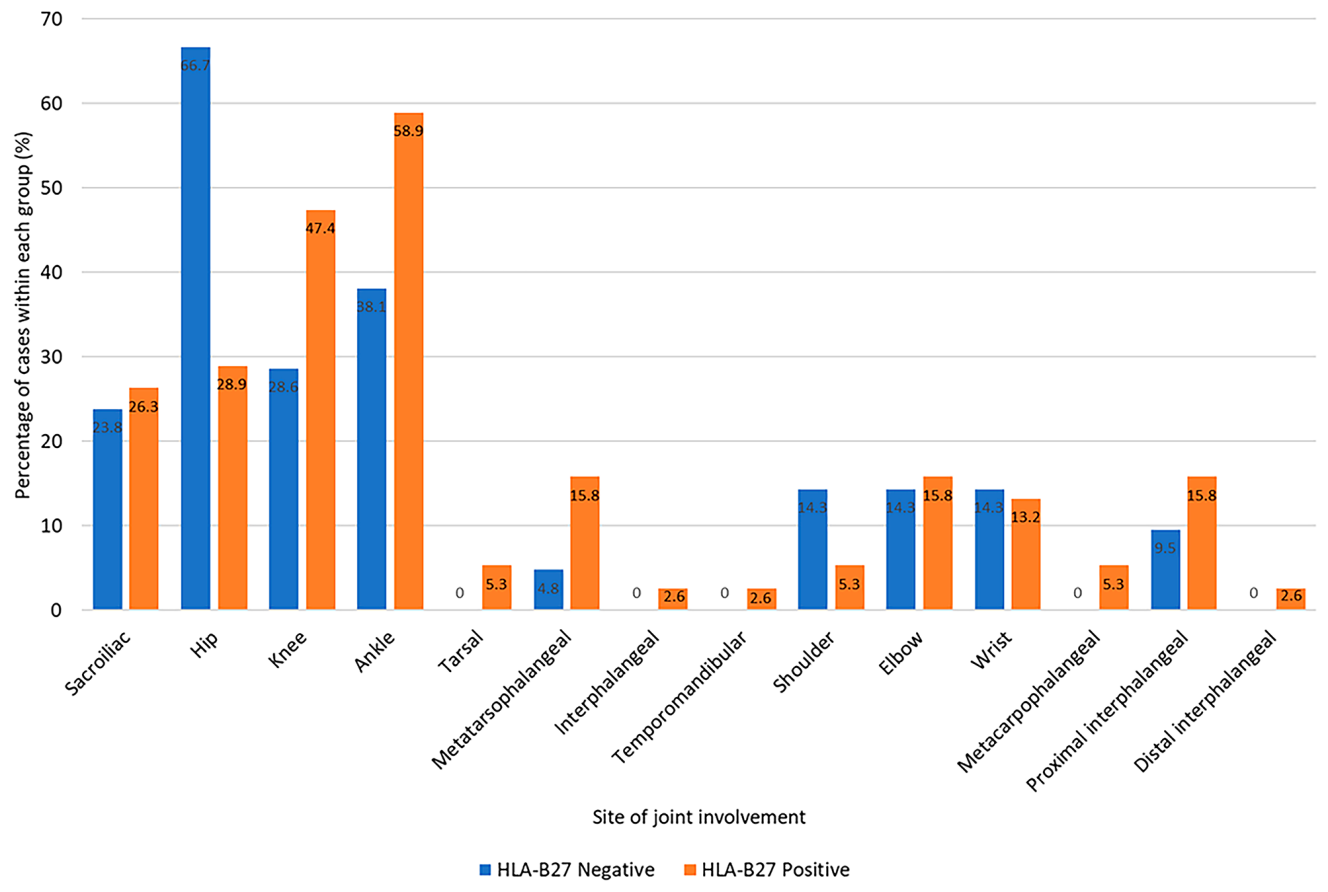
Distributions of joint involvement at the time of diagnosis of enthesitis-related arthritis in HLA-B27 positive and negative patients (*N* = 59)

Most of the patients were males (53 of 59, 89.8%). The mean(± SD) duration of follow-up was 3.9 ± 2.4 years, and the mean ± SD age of onset for patients in this study was 10.8 ± 2.7 years. Positive HLA-B27 was found in 38 patients (64.4%). At diagnosis, a predominant proportion of patients presented with oligoarthritis, characterized by having an affected joint count ranging from 2 to 4 in 24 of 59 (40.7%) followed by polyarthritis, characterized by having an affected joint count of 5 or more in 23 of 59 (39.0%). Within the HLA-B27 positive group, 16 of 38 patients (42.1%) presented with oligoarthritis, while in the HLA-B27 negative group, polyarthritis was more common in 9 of 21 patients (42.9%). Figure 1 shows a comprehensive overview of joint involvement at diagnosis. Ankle joints were the most affected in 30 patients (50.8%), followed by hip joints in 25 patients (42.4%), knee joints in 24 patients (40.7%), and sacroiliitis in 15 patients (25.4%). In the HLA-B27 positive group, the most affected joints were ankle joints in 22 patients (58.9%), knee joints in 18 patients (47.4%), and hip joints in 11 patients (28.9%). In contrast, the HLA-B27 negative group had significantly higher percentage of hip involvement compared to the HLA-B27 positive group (14 of 21 patients, 66.7% vs. 11 of 38, 28.9%, *p* = 0.005).

Enthesitis was present in more than half of the patients at diagnosis (59.3%, 35 of 59). The median number of active enthesitis (IQR) at the time of diagnosis was 1 (0–3) site(s). The most common enthesitis site was the Achilles tendon insertion into calcaneus in 27 patients (45.8%), and the other sites were quadriceps tendon to patella in 13 (22%); plantar fascia insertion into metatarsal head in 11 (18.6%), in calcaneus in 10 (16.9%), and base of metatarsal in 2 (3.4%); infrapatellar ligament insertion to tibial tuberosity in 7 (11.9%) and patella in 3 (5.1%); hip extensor insertion into greater trochanter of femur in 3 (5.1%); common extensor insertion into lateral epicondyle of humerus in 2 (3.4%); and supraspinatus insertion into greater tubercle of humerus in 1 (1.7%). The HLA-B27 positive group had higher percentage of enthesitis at diagnosis compared to the HLA-B27 negative group (68.4% vs. 42.9%), nearly significant (*p* = 0.056). There were significantly higher baseline inflammatory marker levels for both ESR and CRP in the HLA-B27 positive group compared to the HLA-B27 negative group (*p* = 0.001). Regarding CBC, the HLA-B27 positive group also had significantly lower mean hemoglobin and hematocrit levels (*p* = 0.001 and *p* = 0.002, respectively), and higher mean platelet count (nearly significant, *p* = 0.059). Table 1 summarizes the overall baseline clinical characteristics of ERA patients with comparisons between the HLA-B27 positive and negative groups.

**Table 1:** Baseline clinical characteristics of enthesitis-related arthritis patients comparing between HLA-B27 positive and negative groups

Variables	Overall	HLA-B27 positive	HLA-B27 negative	*p*-value
	(*N* = 59)	(*n* = 38)	(*n* = 21)	
Male, n (%)	53 (89.8)	35 (92.1)	18 (85.7)	0.656
Age at onset (years)	10.8 ± 2.7	10.9 ± 2.7	10.6 ± 2.6	0.618
Age at diagnosis (years)	11.2 ± 2.5	11.3 ± 2.6	11 ± 2.5	0.729
Duration of follow-up (years)	3.9 ± 2.4	4.3 ± 2.6	3.2 ± 2	0.112
Family history of HLA-B27 related disease**, n (%)	7 (11.9)	4 (10.5)	3 (15.8)	0.568
Recent infection***, n (%)	7 (11.9)	3 (7.9)	4 (19.0)	0.233
Disease activity at diagnosis				
Active joint count	2 (2–5)	2 (1.8-5)	3 (2–5)	0.438
Enthesitis, n (%)	35 (59.3)	26 (68.4)	9 (42.9)	0.056
Active enthesitis count	1 (0–3)	1 (0–3)	0 (0-2.5)	0.154
Hip arthritis, n (%)	25 (42.4)	11 (28.9)	14 (66.7)	0.005*
Sacroiliitis, n (%)	14 (23.7)	9 (23.7)	5 (23.8)	1.000
Inflammatory back pain****, n (%)	11 (18.6)	7 (18.4)	4 (19)	1.000
Limited joint count	1 (1–2)	1 (1–2)	1 (1-2.5)	0.178
Acute symptomatic uveitis, n (%)	5 (8.5)	4 (10.5)	1 (4.8)	0.646
JSpADA score at diagnosis	3.5 (2.5–4.5)	4 (3-4.5)	3.25 (1.9–4.5)	0.285
Laboratory results at diagnosis				
White blood cell count (cells/mm^3^)	9867.8 ± 2630.3	10229.5 ± 2537.8	9213.3 ± 2729.5	0.157
Neutrophil (%)	57.4 ± 11.7	57.9 ± 10.3	56.4 ± 14.2	0.646
Hemoglobin (g/dL)	11.4 ± 1.7	10.9 ± 1.8	12.4 ± 1.2	0.001*
Hematocrit (%)	36.0 ± 5.0	34.5 ± 5.1	38.6 ± 3.6	0.002*
Platelet (cells/mm^3^)	441118.6 ± 135312.8	465815.8 ± 145753.9	396428.6 ± 102664.3	0.059
Erythrocyte sedimentation rate (mm/h)	41 (20–68)	57 (32-73.5)	19 (7.35-55)	0.001*
C-reactive protein (mg/L)	19.7 (5.9–51)	26.2 (13.3–67)	4.52 (0.65-22)	0.001*

*p < 0.05

**Family history of HLA-B27-related disease in first degree relatives including enthesitis-related arthritis, ankylosing spondylitis, inflammatory bowel disease with sacroiliitis, Reiter syndrome, and acute anterior uveitis

***Recent infection referred to the history of recent infection prior to the onset of enthesitis-related arthritis

****Inflammatory back pain referred to the history or the presence of inflammatory back pain

Abbreviations: JSpADA: Juvenile Spondyloarthritis Disease Activity

### Treatment and disease activity during the follow-up period

Regarding treatment, NSAIDs were the most commonly prescribed drugs (96.6%, 57 out of 59), followed by methotrexate (84.7%, 50 out of 59). Significantly higher use of bridging systemic corticosteroids (oral prednisolone) and anti-TNF were observed in the HLA-B27 positive group compared to the HLA-B27 negative group (*p* = 0.001 and 0.018, respectively). There were also higher utilization of methotrexate, sulfasalazine, and adjunctive intraarticular corticosteroids in the HLA-B27 positive group, although not statistically significant. Regarding the duration of the medication, the use of sulfasalazine had the longest duration in both the HLA-B27 positive and negative groups. Additionally, the duration of methotrexate use in the HLA-B27 positive group was statistically significantly longer, with a median duration (IQR) of 1.7 (1.1–3.1) years (*p* = 0.04). The general treatment details and comparison between both groups are summarized in Table 2.

**Table 2:** Overview of treatment and disease activity during the follow-up period

Variables	Overall	HLA-B27 positive	HLA-B27 negative	*p*-value
	(*N* = 59)	(*n* = 38)	(*n* = 21)	
Treatment medications				
NSAIDS, n (%)	57 (96.6)	36 (94.7)	21 (100)	0.285
Systemic corticosteroids, n (%)	26 (44.1)	23 (60.5)	3 (14.3)	0.001*
Intraarticular corticosteroid injection, n (%)	18 (30.5)	14 (36.8)	4 (19.0)	0.160
Methotraxate, n (%)	50 (84.7)	35 (92.1)	15 (71.4)	0.057
Sulfasalazine, n (%)	37 (62.7)	28 (73.7)	9 (42.9)	0.109
Biological agents (anti-TNF), n (%)	17 (28.8)	15 (39.5)	2 (9.5)	0.018*
Duration of medications use (years)				
NSAIDs use	1.4 (0.7–3.9)	1.5 (0.7–4.2)	1.3 (0.7–2.8)	0.704
Prednisolone use	1.0 (0.7–1.8)	1.0 (0.6–2.9)	1.3 (0.9–1.7)	0.356
Methotrexate use	1.6 (0.9–2.7)	1.7 (1.1–3.1)	1.3 (0.6–1.9)	0.040*
Sulfasalazine use	2.4 (1.4–4.1)	2.8 (1.3–3.6)	1.9 (1.8–2.1)	0.777
Biological agents (anti-TNF) use	1.2 (0.7-2.0)	1.6 (0.7-2.0)	1.3 (0.9–1.7)	0.840
On medication at last visit, n (%)	41 (69.5)	29 (76.3)	12 (57.1)	0.126
Clinical manifestation throughout the follow-up period				
Acute symptomatic anterior uveitis, n (%)	5 (8.5)	4 (10.5)	1 (4.8)	0.646
Accumulative hip arthritis, n (%)	29 (49.2)	14 (36.8)	15 (71.4)	0.011*
Accumulative sacroiliitis	23 (39)	15 (39.5)	8 (38.1)	0.917
Inflammatory back pain, n (%)	19 (32.2)	13 (34.2)	6 (28.6)	0.657
Accumulative active joint count, median (IQR)	4 (2.0–7.0)	3 (2–6)	4 (2-7.5)	0.684
Accumulative active enthesitis count, median (IQR)	2 (0.8-4.0)	2 (1-3.5)	1 (0-4.5)	0.516
Duration from diagnosis to onset of sacroiliitis (years)	0 (0-0.6)	0 (0-3.1)	0 (0–0)	0.205
Duration from diagnosis to hip arthritis (years)	0 (0–0)	0 (0-0.1)	0 (0–0)	0.164
Disease activity				
Relapse disease, n (%)	13 (22)	11 (28.9)	2 (9.5)	0.109
Refractory disease, n (%)	21 (35.6)	16 (42.1)	5 (23.8)	0.160
Active disease at last visit, n (%)	9 (15.3)	5 (13.2)	4 (19)	0.708
JSpADA score at each time point				
At 6 months of follow-up	1.5 (0.5–2.5)	1.75 (0.9-3)	0.5 (0.5–1.7)	0.028*
At 12 months of follow-up	0.5 (0-1.5)	1 (0.4-2)	0.5 (0-0.8)	0.040*
At last visit	0 (0–1)	0 (0-0.5)	0 (0-0.6)	0.768

*p < 0.05

Abbreviations: anti-TNF: anti-tumor necrosis factor; NSAIDs: nonsteroidal anti-inflammatory drugs; JSpADA: Juvenile Spondyloarthritis Disease Activity

For clinical characteristics throughout the treatment period, the appearance of hip arthritis from first diagnosis to the last visit was significantly higher in the negative HLA-B27 group compared to the positive HLA-B27 group (15 of 21, 71.4% vs. 14 of 38, 36.8%, *p* = 0.011). The percentage of accumulated hip joint involvement increased from baseline in both groups. There were also higher percentage of patients with sacroiliitis, inflammatory back pain, and acute symptomatic uveitis in the HLA-B27 positive group than in the HLA-B27 negative group, without statistical significance. There were no discernible differences in relapse and refractory disease between the two groups classified according to HLA-B27 status in the overall disease course. The details of the clinical characteristics and disease course during the follow-up period are shown in Table 2. No patients were diagnosed with inflammatory bowel disease during the clinical course.

**Fig. 2:**
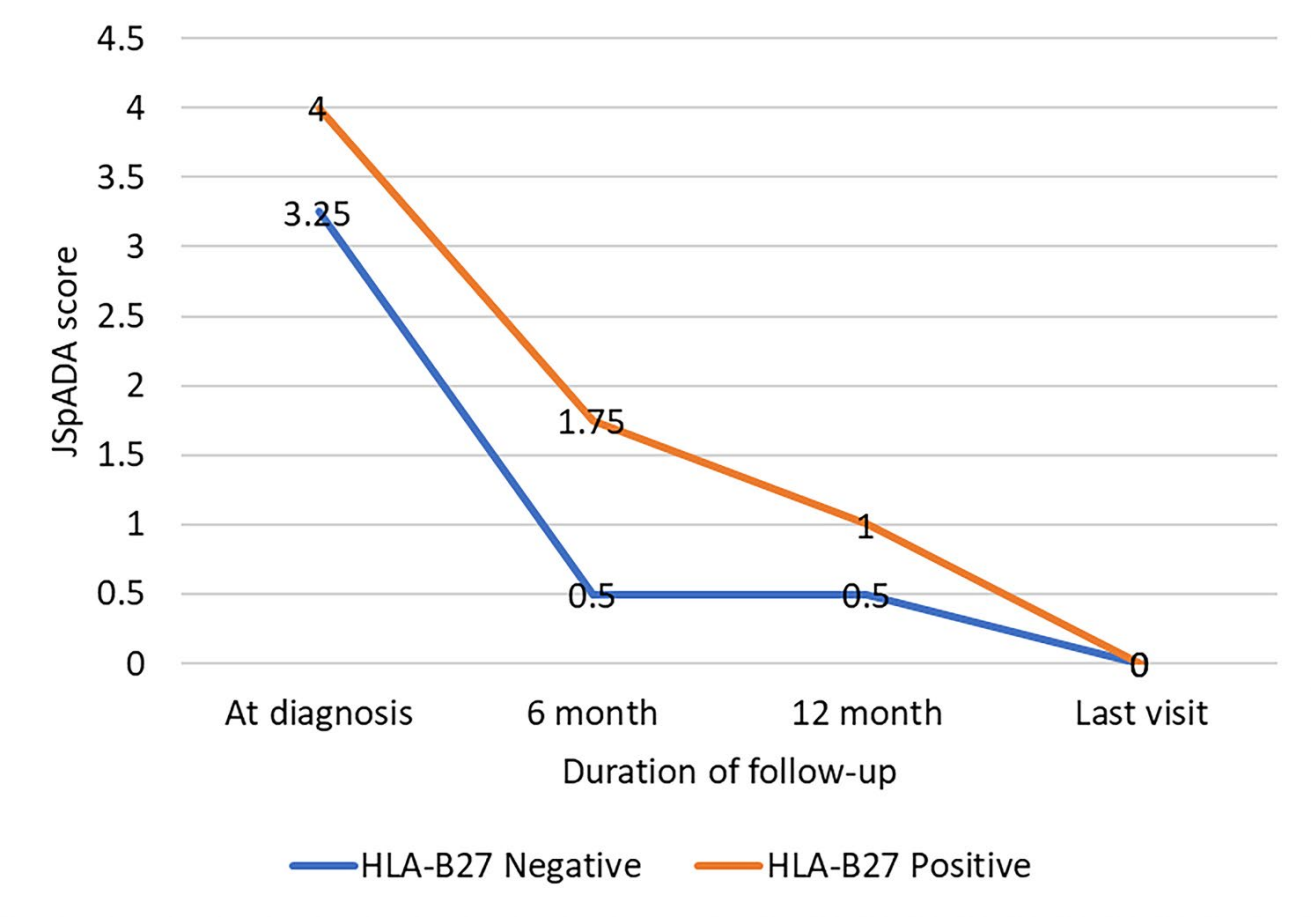
Trends in median scores for Juvenile Spondyloarthritis Disease Activity (JSpADA) assessed at 0, 6, 12 months, and the last visit in patients with HLA-B27 positive and negative enthesitis-related arthritis

In evaluating treatment outcomes using the JSpADA score as a measure of disease severity, there were no differences in scores between the two groups at the time of initial diagnosis. However, a significant difference was observed in the HLA-B27 positive group compared to the HLA-B27 negative group, revealing median (IQR) scores of 1.75 (0.9-3) vs. 0.5 (0.5–1.7) at 6 months (*p* = 0.028) and 1 (0.4-2) vs. 0.5 (0-0.8) at 12 months (*p* = 0.04), as shown in Table 2. The trend is depicted in Fig. 2, illustrating an overall downward trend in the median JSpADA scores in both groups from diagnosis to the last visit. In particular, in the HLA-B27 negative group, the JSpADA scores demonstrated a reduction of more than 80% from diagnosis to 6 and 12 months of follow-up. At the same time, the HLA-B27 positive group showed a reduction of 50% at 6 months and a reduction of 75% at 12 months from the time of diagnosis. However, both groups reached a median JSpADA score of zero at their last visit.

**Fig. 3:**
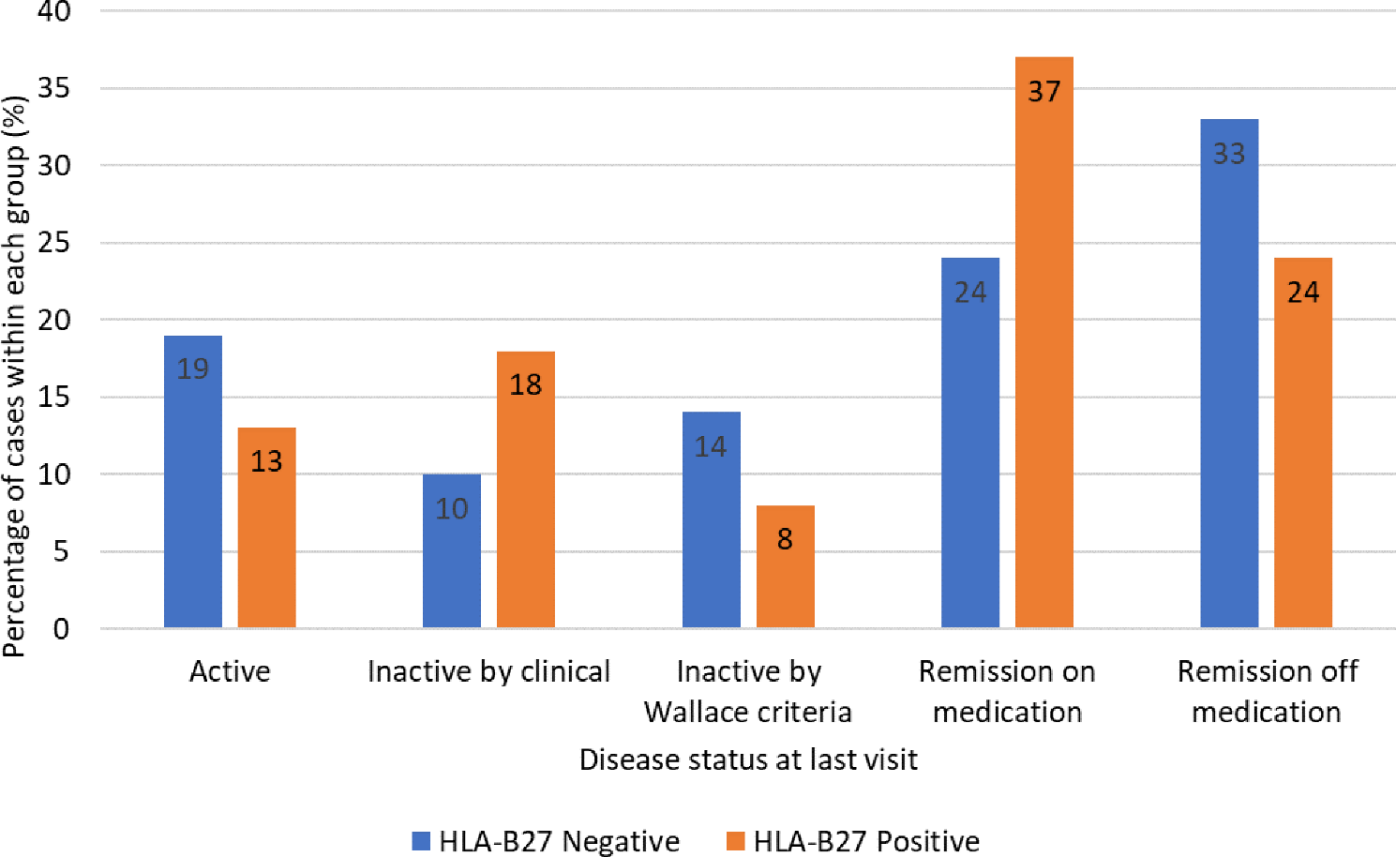
Disease status at the last visit in HLA-B27 patients with positive and negative enthesitis-related arthritis (*N* = 59)

At the last visit, among HLA-B27 positive patients, the majority achieved remission on medication, accounting for 37% (14 of 38 patients), while among HLA-B27 negative patients, the majority achieved remission without medication, representing 33% (7 of 21 patients). Figure 3 elaborates the disease status of patients with ERA at the last visit by comparing the positive and negative HLA-B27 groups, shown as percentage within each group.

The ASAS classification criteria were applied to ERA patients older than 16 years at the last visit, which included a total of 23 patients. Of this, 13 of 23 (56.5%) met the criteria for peripheral spondyloarthritis (SpA) and 10 of 23 (43.5%) met the criteria for axial SpA. Among those who met the peripheral SpA criteria, 10 of 13 patients had positive HLA-B27 as one of the SpA characteristics. For those who met the axial SpA criteria, 9 of 10 patients had positive HLA-B27.

## Discussion

Our study explored demographic data, clinical characteristics at baseline and disease course, treatment, disease activity, and outcomes in ERA patients. HLA-B27 positive patients (64.4%) had significantly higher levels of inflammatory markers, lower hemoglobin and hematocrit at diagnosis, greater use of bridging systemic corticosteroids and anti-TNF, longer duration of methotrexate use, and higher JSpADA scores at 6 and 12 months of treatment. On the contrary, HLA-B27 negative patients had significantly more hip arthritis at both baseline and during the disease course.

The prevalence of HLA-B27 in our ERA patients was 64.4%, consistent with studies from another tertiary center in Thailand, Singapore, China, Sweden, and the United States (US), where more than 50% of the patients were HLA-B27 positive [14–16, 25–27]. On the contrary, a study from France reported HLA-B27 positivity less than 50% [28], while a study from Taiwan documented positivity up to 97% [29]. This indicates potential ethnic variability as a contributing factor to the differences in HLA-B27 positivity [14–16, 25–30]. The percentage of family history in first-degree relatives with HLA-B27-related disease in our study (11.9%) was consistent with previous studies ranging from 11-15.4% [14, 26, 29]. However, higher prevalences of family history were reported in France (28%) [28] and China (27.4%) [25], in contrast to the aforementioned studies.

In our study, most of the patients were males (89.8%), with a mean age at the time of onset of 10.8 ± 2.7 years. Comparisons with previous cohort studies revealed similar median onset ages ranging from 9.5 to 11.6 years [14, 16, 25, 26, 29]. However, a previous study reported a later median age onset of 14 years [28]. Most of the patients at diagnosis presented with oligoarthritis (40.7%), aligned with the findings of previous cohorts [14, 16, 26, 29]. The distribution of the joint at diagnosis predominantly involved peripheral joints in the lower extremities, with the most affected joint in our study being the ankle (50.8%), followed by the hip joint (42.4%) and the knee joint (40.7%), in which these joints also emerged as the top three affected joints in previous studies, although in a different order; in the Singapore study, the hip joint was the most common (43.8%), followed by the knee (37.7%) and the ankle joint (26%); in another tertiary center in Thailand, France and the US, the knee was the most common [14, 16, 26, 28].

When comparing two groups based on HLA-B27 status, our study reveals significantly higher inflammatory markers (ESR, CRP), with lower hemoglobin and hematocrit at diagnosis in the HLA-B27 positive group. The results of a previous multicenter study in the US showed more inflammation occurring in the HLA-B27 positive group compared to the negative group [26]. Interestingly, a study from China which classified ERA patients into those with and without fever reported that ERA patients with fever showed more active disease at the beginning with more anemia and thrombocytosis [24]. The trends of anemia and thrombocytosis in the HLA-B27 positive group could be explained by anemia of inflammation and reactive thrombocytosis. Therefore, these findings from our study indicate more inflammation in the HLA-B27 positive group compared to the negative group, which was consistent with previous studies [25, 26]. At diagnosis, our study identified slightly higher frequencies of enthesitis, inflammatory back pain, acute symptomatic uveitis and the JSpADA score at diagnosis, as well as higher frequencies of sacroiliitis, inflammatory back pain, and acute symptomatic uveitis during follow-up in the HLA-B27 positive group, although without statistical significance, possibly due to the limited number of participants. A larger multicenter study in the US demonstrated a significant increase in active joint counts, sacroiliitis, and the JSpADA score at diagnosis among the HLA-B27 positive group [25]. Additionally, a study from Singapore showed significantly higher incidences of sacroiliitis at diagnosis and the course of treatment in the HLA-B27 positive group, also having higher incidences of uveitis, active joint count, and enthesitis in the HLA-B27 positive group, although without statistical significance [14]. Therefore, our results emphasize a potential trend toward higher disease activity and presence of associated findings such as sacroiliitis and uveitis in the HLA-B27 positive group compared to the HLA-B27 negative group.

In addition to HLA-B27 positivity, individuals with hip arthritis and sacroiliitis had been recognized to have poor prognostic factors [15, 31]. Our data surprisingly revealed a significantly higher incidence of hip arthritis both at diagnosis and throughout the course of treatment in the negative group for HLA-B27. Similarly, Singapore’s findings showed a slightly higher number of hip arthritis occurrences in the HLA-B27 negative group compared to the HLA-B27 positive group at diagnosis (50% vs. 42.5%) and during the follow-up period (69.2% vs. 58.3%) for the negative and positive groups of HLA-B27, respectively, although without statistical significance [14]. In contrast, findings from another tertiary center in Thailand showed that HLA-B27 positive patients had more hip involvement than HLA-B27 negative patients (46% vs. 18.8%) [16]. However, considering the occurrence of hip arthritis, particular attention should be paid to the observation of hip arthritis even in the negative HLA-B27 group for early detection and proper treatment.

According to standard treatment recommendations, NSAIDs are considered the primary treatment of DMARD, and systemic corticosteroids can be considered as bridging therapy [13]. Our study showed that NSAIDs were the most utilized drugs, followed by methotrexate, sulfasalazine, and prednisolone. While anti-TNF is recommended for sacroiliitis [13], resource limitations, particularly limited access and high cost of biologic drugs [13], lead to alternative combinations of DMARDs and NSAIDs in some countries [7, 31, 32], including Thailand [7, 32]. In Thailand, the eligibility criteria for anti-TNF for JIA are considered based on individual health benefit schemes and are indicated in patients with refractory disease despite treatment with combinations of NSAIDs, DMARDs, and systemic corticosteroids [7]. In our study, 28.8% of ERA patients met the indication for anti-TNF, and HLA-B27 positive individuals were significantly more likely to be prescribed it compared to HLA-B27 negative groups. This trend also existed for systemic corticosteroids as a bridging therapy for patients with higher disease activity, which was significantly higher in HLA-B27 positive individuals compared to HLA-B27 negative groups. It should be noted that the duration of systemic corticosteroids used in our study was longer than the standard recommendations [20], to help control the disease in the early treatment phase, mainly for patients who may need biologics but had limited access due to financial problems or did not meet the indicated criteria according to the health benefit schemes. However, we acknowledge that the use of systemic corticosteroids may lead to disease damage as it is the predictor of extraarticular damage [7] and is associated with a suboptimal quality of life in JIA [5]. Furthermore, previous studies found a significantly higher incidence of anti-TNF use in HLA-B27 positive individuals compared to negative groups [14]. Our study also showed that the duration of methotrexate was significantly longer in the HLA-B27 positive group, possibly due to the higher disease activity that required a prolonged period of medication to achieve disease control until remission.

Disease activity, monitored by the JSpADA score, did not show significant differences at diagnosis, but was significantly higher in the HLA-B27 positive group at 6 and 12 months of follow-up. In the HLA-B27 negative group, the improvement in the JSpADA score was greater than in the HLA-B27 positive group, leading to a median score of 0 at the last visit in both groups. This was consistent with the study by Vilaiyuk et al. [6], which demonstrated that HLA-B27 negative patients showed more improvement than those with HLA-B27 positive in the first 3 months of the follow-up period, potentially indicating that the HLA-B27 positive group had a more refractory course of the disease. Our study also showed a higher proportion of refractory disease in HLA-B27 positive patients, although without statistical significance. At the last visit in our study, active disease was observed in 15.3% and HLA-B27 positivity did not significantly affect the outcome at the last visit, similar to the study by Arkachaisri et al. [14]. This could be attributed to HLA-B27 positive patients, despite having a higher disease activity score, receiving more systemic corticosteroids and anti-TNF compared to the HLA-B27 negative group. Consequently, the disease outcome at the last visit did not differ between the two groups. Among patients with ERA who turned to adulthood, the application of ASAS criteria showed the prevalence of peripheral SpA in more than half of the patients, highlighting that patients with the SpA spectrum who developed clinical manifestations earlier from childhood exhibit more peripheral joint involvement. Additionally, younger age of onset was associated with HLA-B27 positivity in Thai adults with axial SpA [18]. Table 3 summarizes the cohorts of patients with ERA from various countries with comparisons with the present study [14, 16, 25–31, 33, 34]. The results of our study add new information to the previous Thai cohort by highlighting the differences between HLA-B27 positive and negative ERA patients in the aspects of clinical characteristics at diagnosis and follow-up, disease activity, medications, and outcome, providing data specifically for both groups and overall patients, as well as the application of ASAS classification criteria in patients who turned into adults with ERA to identify the distinct phenotype, which can provide useful information for adult rheumatologists during transition and long-term follow-up.

**Table 3:** Summary of ERA cohorts from various countries with comparison to the present study

Author	Year	Country	N	% of total JIA	Positive HLA-B27 (%)	Age at onset (years)*	Sacro-iliitis (%)	Enthesitis (%)	Uveitis (%)	Hip arthritis (%)	Most affected joint (%)	Systemic cortico-steroids (%)	Sulfa-salazine (%)	Metho-trexate (%)	Anti-TNF (%)
Jittawattanarat	2024	Thailand	59	33.1	64.4	10.8 ± 2.7	23.7	59.3	8.5	42.4	Ankle (50.8)	44.1	62.7	84.7	28.8
(present study)															
Chan [30]	2023	Hong Kong	41	40	95	12(10.3–15)^a^	78	NA	NA	34.1	Sacroiliitis (78)	NA	NA	NA	29
Vilaiyuk [16]	2022	Thailand	66	NA	75.8	10.2 ± 3.1	18.6	43.9	3	40.9	Knee (62.1)	NA	78.8	93.9	59
Naveen [31]	2021	India	73	NA	98	14(11–15)	59	90	1.78	31.5	Sacroiliitis (59)	NA	NA	NA	2.7
Arkacharisri [14]	2021	Singapore	146	30.8	82	11.9(9.4–14.0)	39.7	24	3.4	43.8	Hip (43.8)	52.1	78.8	77.4	72.6
Shih [29]	2019	Taiwan	73	39.9	97	11 ± 3.2	16	97	10	NA	NA	53	62	74	78
Zanwar [27]	2018	India	127	NA	86.5	14.3 ± 2.4 ^b^	43	NA	NA	12.5	NA	NA	3.9	15.8	0
Goirand [28]	2018	France	114	NA	43	9.6(2.7)	29	72	NA	NA	Knee (58)	NA	NA	NA	NA
Gmuca [26]	2017	USA	234	NA	59.2	11.6(9.8–13.7)^a^	55.6	75.2	5.6	19.2	Knee (45.7)	14.6	NA	NA	15
Guo [25]	2015	China	146	NA	58.9	10.3^a^	43.8	37	7.5	35.6	Knee (45.2)	NA	NA	NA	68.5
Srivastava [34]	2015	India	107	NA	79	12(4–16)	21	59	12	NA	NA	NA	NA	NA	NA
Demirkaya [33]	2011	Turkey	120	18.9	63.3	15.3 ± 2.8^b^	NA	NA	6.7	NA	NA	NA	100	NA	NA

*Displayed as mean ± standard deviation or median (interquartile range)

^a^Age at diagnosis

^b^Age at enrollment

Abbreviations: ERA: enthesitis-related arthritis; JIA: juvenile idiopathic arthritis; NA: not applicable

Although our study did not have any patients who developed inflammatory bowel disease during the follow-up period, this potential association should be concerned in patients with ERA or juvenile spondyloarthropathy especially those with positive HLA-B27 [19]. As mentioned earlier that ERA patients with positive HLA-B27 were more anemic and had higher inflammatory markers at baseline, this group of patients may need close monitoring for development of possible inflammatory bowel disease and require further work-up. For clinical implications, we propose that in ERA patients with positive HLA-B27, those exhibiting higher inflammatory markers at baseline may need further work-up for possible inflammatory bowel disease. Moreover, in ERA patients with positive HLA-B27, routine magnetic resonance imaging should be performed for early detection of active disease with closer monitoring and more aggressive escalation of therapy when indicated.

There are some limitations in our study. First, this study was a retrospective review of an electronic medical record. Although the rheumatology clinic had a routine record form for every visit, it is inevitable that there may be missing data at certain follow-up time points. Second, as there was a limited number of cases in our study, this may have affected subgroup comparisons and may not have been sufficient to achieve statistical significance. Third, since this was a single-center study in Thailand, it may not have represented the entire ERA population. A larger prospective multicenter study is recommended to address these limitations. However, this study was conducted in the largest nationwide referral center that represented real-life practice in a developing country, using JSpADA scores to assess disease activity and response to treatment, long-term follow-up to explore the natural course of disease, and application of ASAS criteria to classify ERA patients during adulthood, which could provide useful information for clinicians providing patient care in the global community.

## Conclusion

This study demonstrates that HLA-B27 positive ERA patients exhibited higher disease activity and higher disease activity scores within the first year of treatment compared to the HLA-B27 negative group. This led to higher utilization of bridging systemic corticosteroids, anti-TNF, and longer duration of DMARDs, to effectively control the disease in HLA-B27 positive ERA patients until remission. Our findings raise awareness of the higher disease burden among ERA patients with positive HLA-B27 in real world practice. More multicenter prospective studies should be conducted to explore these aspects of clinical practice in the global community.

## Data Availability

The datasets used and/or analysed during the current study are available upon reasonable request by contacting the corresponding author.

## Abbreviations

anti-TNF: antitumor necrosis factor
ASAS: Assessment of SpondyloArthritis international Society
CBC: complete blood count
CRP: C-reactive protein
DMARDs: disease-modifying antirheumatic drugs
ERA: enthesitis-related arthritis
ESR: erythrocyte sedimentation rate
HLA-B27: human leukocyte antigen B27
ILAR: International League of Associations for Rheumatology
IQR: interquartile range
JIA: juvenile idiopathic arthritis
JSpADA: Juvenile Spondyloarthritis Disease Activity
NSAIDs: non-steroidal anti-inflammatory drugs
SD: standard deviations
VAS: visual analog pain scale
